# Adaptive learning from outcome contingencies in eating-disorder risk groups

**DOI:** 10.1038/s41398-023-02633-w

**Published:** 2023-11-04

**Authors:** Alexandra C. Pike, Ann L. Sharpley, Rebecca J. Park, Philip J. Cowen, Michael Browning, Erdem Pulcu

**Affiliations:** 1 Department of Psychology and York Biomedical Research Institute, University of York, Heslington, York, YO10 5DD UK; 2 Anxiety Laboratory, Neuroscience and Mental Health Group, Institute of Cognitive Neuroscience, University College London, 17-19 Queen Square, London, WC1N 3AR UK; 3 Department of Psychiatry, University of Oxford, Warneford Hospital, Oxford, OX3 7JX UK; 4 Oxford Health NHS Foundation Trust, Warneford Hospital, Oxford, UK

**Keywords:** Psychiatric disorders, Neuroscience

## Abstract

Eating disorders are characterised by altered eating patterns alongside overvaluation of body weight or shape, and have relatively low rates of successful treatment and recovery. Notably, cognitive inflexibility has been implicated in both the development and maintenance of eating disorders, and understanding the reasons for this inflexibility might indicate avenues for treatment development. We therefore investigate one potential cause of this inflexibility: an inability to adjust learning when outcome contingencies change. We recruited (*n* = 82) three groups of participants: those who had recovered from anorexia nervosa (RA), those who had high levels of eating disorder symptoms but no formal diagnosis (EA), and control participants (HC). They performed a reinforcement learning task (alongside eye-tracking) in which the volatility of wins and losses was independently manipulated. We predicted that both the RA and EA groups would adjust their learning rates less than the control participants. Unexpectedly, the RA group showed elevated adjustment of learning rates for both win and loss outcomes compared to control participants. The RA group also showed increased pupil dilation to stable wins and reduced pupil dilation to stable losses. Their learning rate adjustment was associated with the difference between their pupil dilation to volatile vs. stable wins. In conclusion, we find evidence that learning rate adjustment is unexpectedly higher in those who have recovered from anorexia nervosa, indicating that the relationship between eating disorders and cognitive inflexibility may be complex. Given our findings, investigation of noradrenergic agents may be valuable in the field of eating disorders.

## Introduction

Eating disorders (EDs) are a cluster of psychiatric disorders characterised by altered eating attitudes and behaviours, alongside over-valuation of the control of eating, weight and/or shape [1]. EDs are relatively common [2–4], often severely disabling [5], and can become chronic [6, 7], with high rates of mortality [3, 8, 9] and relatively low rates of treatment response or remission [10–13]. Psychological treatments can help, but only in some cases [14], and there are few efficacious pharmacological treatments [15]. To improve treatment success, greater knowledge of the cognitive differences that precipitate and maintain the ritualistic, rigid behaviours that characterise many EDs would be valuable [16]. Furthermore, understanding cognitive mechanisms underlying a disorder may combine with our knowledge of the actions of pharmacological agents on those mechanisms to indicate new treatment directions.

‘Cognitive inflexibility’ is frequently observed in EDs [17, 18], and can be defined as an inability to adjust or adapt cognitive functions (e.g. learning, and decision-making) in response to changes in the requirements of the task, outcome contingencies, or the goals that the individual is pursuing. This is often probed using set-shifting tasks such as the Wisconsin Card Sort Task [19], where the ‘rule’ that governs correct behaviour changes without warning. On this and similar tasks, those with EDs show worse set-shifting performance, broadly indicating difficulty in flexibly altering responses given changes in the requirements of the task [20]. This is thought to be present as a ‘trait’, i.e. not just a product of the disease-state [21, 22].

‘Set-shifts’ induce unexpected uncertainty or volatility – changes in the underlying probabilistic structure that has been learnt by the individual [23, 24]. This type of uncertainty is common in everyday life. For example, you may have a local restaurant you really like but have recently found the food to be less good than it had been. Where you go to eat in the future will depend on whether the recent bad meals occurred by chance (in which case you should continue going to the same restaurant), or whether there has been a reduction in the underlying quality of the food (i.e. the quality is volatile, in which case you should switch restaurant). The question addressed in this paper is whether people with EDs are able to perceive and change their behaviour appropriately in response to outcome volatility, and whether difficulties with this might underpin neuropsychological findings of poor cognitive flexibility.

Recent work in this area has used a task [25] in which the volatility of outcomes is manipulated independently between blocks. Computational modelling allows us to estimate a parameter referred to as a 'learning rate', which can be understood as the extent to which each outcome influences the learnt value of particular options. When outcomes are volatile, learning rates should be higher, as more recent outcomes are more predictive of the actual value of an option than outcomes that occurred further back in history. Healthy participants performing this task are able to adjust their learning rate in response to volatility in an approximately optimal way [26]. A recent adaptation of this task has shown that participants are also able to maintain separate estimates of different valences of outcomes (‘wins’ and ‘losses’), and track the volatility of each of these, adjusting learning rates for wins and losses independently [25]. Importantly, using computational modelling allows us to adjudicate between competing hypotheses regarding poor set-shifting performance in eating disorders: greater noise in behavioural decision-making, generally reduced learning rates, or reduced learning rate adjustment in response to volatility.

We hypothesised that adjustment in learning rates in response to volatility would be reduced in individuals with EDs, in line with the cognitive neuroscience evidence suggesting that those with EDs may experience difficulties with cognitive flexibility, and struggle to adjust their behaviour [17, 20–22]. A finding that those in ED groups adjusted their learning rates less would also correspond with a similar finding in anxiety disorders [27], which would be unsurprising given the high comorbidity between anxiety disorders and EDs [28].

Biologically, noradrenaline (NA) may signal unexpected uncertainty [24]. It is possible to indirectly measure the response of the central noradrenergic system using pupillometry [29, 30]; phasic changes in pupil diameter have been observed to correlate with volatility [25, 27, 29], and pupil dilation may also be linked to surprise [31]. Pharmacological manipulations that increase the release of NA are able to improve performance on (attentional) set-shifting tasks in rats [32]. Furthermore, NA deafferentation in rats impairs set-shifting performance [33]. In humans, propranolol, which attenuates NA transmission, reduces volatility-related increases in learning rates [34]. However, not all of the evidence paints such a clear picture of the role of NA in signalling unexpected uncertainty. Jepma et al. [35] found that atomoxetine (a NA transporter blocker) caused an increase in learning rate after an alteration in outcome contingencies if the baseline learning rate was low, but otherwise, atomoxetine caused a reduction in learning rate. There has been early evidence for the efficacy of atomoxetine in the treatment of binge-eating disorder, and anorexia nervosa with binge-purge features [36, 37]. Additionally, research has indicated that there is reduced NA functioning in ED patients, including those who have recovered [38–40]. We therefore recorded pupil dilation to examine whether any changes in learning rate adjustment were reflected in altered pupil dilation changes, which could in turn reflect differences in noradrenergic transmission. We hypothesised that corresponding to a reduction in learning rate adjustment, those with EDs might show a reduced pupil dilation response to volatility. This may indicate a lack of sensitivity to changes in outcome volatility.

## Methods and materials

This study was preregistered on clinicaltrials.gov, with the identifier NCT03450291. This study was approved by the University of Oxford’s Central University Research Ethics Committee (reference R51898). Open data and code are not available as not all participants consented to have their data shared, even if they could not be identified after anonymisation.

### Participants

Three groups of female participants, selected to cover a range of different ED-relevant phenotypes, were recruited (82 in total): those who had recovered from anorexia nervosa (RA, *n* = 25), those who were highly concerned about their eating, shape and weight, but did not have a formal ED diagnosis (EA, *n* = 25), and control participants who had never had an eating disorder and were below various markers on self-report questionnaires about eating, shape and weight (HC, *n* = 32). The criteria for each of these groups, along with further details of the inclusion and exclusion criteria, may be found in the Supplementary Material.

### General procedure

The study involved the completion of pre-screening questionnaires: the Eating Attitudes Test (EAT-26; [41]), Eating Disorders Examination Self-Report Questionnaire (EDE-Q; [42]), and Clinical Impairment Assessment for eating disorders (CIA; [43]). If eligible, participants were invited for a single study visit. During that visit, the Structured Clinical Interview for the Diagnostic and Statistical Manual of Mental Disorders, Fifth Edition (DSM-5) (Revised Version) was performed. Subsequently, participants completed additional self-report questionnaires online (see Table 1). They then completed the learning task [25] described below, alongside eye-tracking.

**Table 1:** Demographic details.

	EA	RA	HC	Group difference
Age	23.78 (5.15)	23.48 (3.77)	25.03 (6.38)	None (*p* = 0.469)
BMI	21.76 (2.78)	21.79 (2.43)	23.09 (4.81)	None (*p* = 0.123)
EDE-Q	3.16 (0.77)	1.49 (1.23)	0.99 (0.84)	*F*[2,79] = 36.37, *p* < 0.001
EAT-26	28.52 (6.80)	7.43 (12.21)	5.33 (5.00)	*F*[2,79] = 61.39, *p* < 0.001
CIA	19.52 (9.02)	9.09 (8.84)	3.73 (4.11)	*F*[2,79] = 32.69, *p* < 0.001
BDI-II	8.39 (9.39)	5.61 (5.68)	2.60 (4.65)	*F*[2,79] = 5,74, *p* = 0.005
STAI trait	38.74 (13.21)	40.26 (9.83)	29.63 (8.03)	*F*[2,79] = 8.77, *p* < 0.001
STAI state	32.83 (12.65)	31.74 (5.44)	27.53 (9.45)	None (*p* = 0.103)
OCI-R	7.65 (7.37)	6.78 (6.63)	5.40 (6.57)	None (*p* = 0.428)

Summary of participant demographics and questionnaire measures.

Mean, standard deviation scores, and ANOVA results (with 3 levels of ‘group’) are displayed.

*BMI* Body Mass Index, *EDE-Q* Eating Disorders Examination (Self-Report Questionnaire), *EAT-26* Eating Attitudes Test, 26 item version, *CIA* Clinical Impairment Assessment, which measures the extent to which eating disorder symptoms affect one’s daily life, *BDI-II* Beck’s Depression Inventory (version 2), *STAI* Spielberger Stait-Trait Anxiety Inventory, which is divided into two subscales, measuring trait and state anxiety, *OCI-R* Obsessive-Compulsive Inventory (Revised Version).

There was a significant group difference in EDE-Q, EAT-26, CIA, BDI-II and STAI trait, and no significant group differences in STAI state, OCI-R, age or BMI.

### The volatility task

**Fig. 1:**
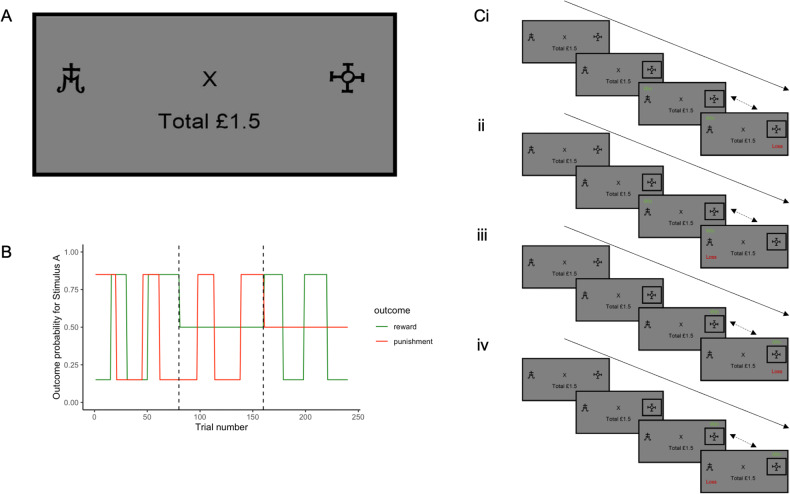
Volatility task details. **A** An example stimulus presentation screen (prior to choice), showing the two stimuli (abstract shapes, referred to as ‘A’ and ‘B’ throughout for convenience), a fixation cross in the centre of the screen, and the participant’s monetary total below the fixation cross. Their monetary total was initialised at £1.50. The two abstract stimuli presented were changed between blocks (i.e. after 80 trials, and after 160) and the same pair of stimuli was always presented within a block, though the side of the screen they appeared on was counterbalanced. Participants were encouraged to take breaks in between blocks. After participants made a choice, they could receive wins or losses. The stimulus-outcome associations for one stimulus, referred to here as stimulus A, are shown in panel (**B**). The associations were exactly reversed for the other stimulus presented in that block – i.e. for stimulus B, the probability of a win is 1 minus the probability of a win for shape A. If, on any given trial, ‘win’ feedback was shown for one stimulus, it could not be shown for the other. However, the summed probability of both outcomes for a given shape, i.e. win + loss, was not 1 as wins and losses were independent. Note that in block 1 (up to trial 80, marked with a dotted line) both win and loss outcomes are volatile, and change between a high probability (0.85) and a low probability (0.15). In blocks 2 and 3 (trials 81:160 and 161:240; order counterbalanced between participants), one outcome was stable (0.5 probability of that outcome resulting from choosing the relevant stimulus), and one was volatile (changing between 0.85 and 0.15 probability of outcome receipt). When outcomes were volatile, the probability of each outcome changed with a frequency of between 14 and 30 trials. The total probability within a block that wins and losses were associated with any given stimulus averaged 50%, such that the task did not systematically favour either of the shapes within a block. As can be noted from panel B, it is possible for a stimulus to be associated with both win and loss, neither win or loss, win only, or loss only. These four possible outcomes are shown as example trial sequences in panel (**C**). The box around shape B reflects the choice the participant made on that trial (so, in this instance, they selected shape B). Regardless of which stimulus was chosen, the outcomes associated with both stimuli were shown. From top to bottom, these are (i) win associated with shape A and loss associated with shape B, (ii) both win and loss associated with shape A, (iii) both associated with shape B, and (iv) win associated with shape B, and loss associated with shape A. Note that these example sequences show the win outcome being displayed before the loss outcome: in the actual task, the order of presentation of these two outcomes was counterbalanced (reflected by the dotted arrows), and there was a jittered delay of between 2 and 6 s before the other outcome was presented. A win outcome resulted in an addition of 15p to their monetary total; a loss resulted in 15p being deducted from their total. Notably, win and loss outcomes were independent, such that knowing the stimulus associated with the win was not informative about the location of the loss. Participants were required to learn over time which stimulus was associated with wins and losses and were asked to aim to maximise their wins and minimise their losses.

This study utilises a task which has been shown to produce adaptive learning in human participants [25–27]. The volatility of outcomes (here defined as the frequency at which the associations between the stimuli and outcomes alter) is manipulated between blocks (Fig. 1). In brief, the task consisted of three blocks (each consisting of 80 trials), and on any trial, two stimuli were presented (kept constant within blocks), and associated with win and/or loss outcomes. Participants learned using trial and error to select the stimulus that was associated with wins and avoid the stimulus associated with losses. Importantly, the associations between stimuli and outcomes were not necessarily fixed: they could vary within a block (i.e. were ‘volatile’). A stimulus could be associated with both win and loss outcomes on a given trial (or only one of the two outcomes, or neither): therefore, we were able to independently manipulate the volatility of wins and losses between the three blocks. Participants generally display an elevated learning rate for volatile outcomes [26, 27], akin to overweighting more recent outcomes compared to more distal outcomes, and participants have been shown to adjust their learning rates for wins and losses separately as their volatility changes [27]. The reader should note that wherever we analyse behaviour or pupil dilation in response to volatility, we include data from both the first block (block 1) and the block where only that outcome was volatile (i.e. the ‘win volatile’ block for wins, and the ‘loss volatile’ block for losses), to maximise our power to detect differences.

### General statistical approach

Wherever applicable we used a Greenhouse-Geisser correction to adjust for lack of sphericity. To further clarify significant effects from the mixed ANOVAs, we used post-hoc Welch’s t-tests, which conservatively assume unequal variance between groups. We did not correct for multiple comparisons in these analyses: the primary (adjustment of learning rate) and secondary outcomes (valence-specific effects in learning rate adjustment, and differences in pupil dilation under volatility) were pre-registered, and all other analyses are exploratory and designed to aid the interpretation of the results.

### Behavioural analysis

#### Data quality

Participants who showed no evidence of learning during the task (those whose total money won was the same or less than the £1.50 they started with) were removed from further analysis (*n* = 4; comprised of two HC participants, and one from each of the other two groups).

#### Switch/stay analysis

The trial-by-trial tendency of participants to ‘switch’ (i.e. choose a different stimulus on the next trial) or ‘stay’ (i.e. repeat their choice on the next trial) may provide some insight into participants’ behaviour in this volatility task. Those who heavily weigh the most recent outcome against the long-run probability of each outcome associated with each stimulus are more likely to choose to ‘switch’ after a loss and a ‘stay’ after a win. In general, we expected that participants would ‘switch’ more after a negative outcome in blocks where losses are volatile, as the receipt of a loss would indicate a potential change in loss probability. Similarly, we would expect participants to ‘stay’ more after a rewarding outcome in blocks where wins are volatile. Cognitive inflexibility, in particular reduced adjustment to changing outcome probabilities (which we expected to observe in both the RA and EA groups), may manifest as a reduction in this pattern of choices.

We, therefore, analysed the proportion of times that participants chose to switch after receiving an outcome when that outcome was either volatile or stable. We used a logit transform on the proportion of times that participants chose to stay after each outcome to ensure that this was on the real, infinite number line. We subsequently used a repeated-measures ANOVA, with between-subject effects of group and block presentation order and within-subject effects of outcome volatility (volatile vs. stable) and valence of outcome (win vs. loss), to investigate whether there were any between-group differences.

#### Reinforcement learning analysis

The switch/stay analysis described above relies on summary statistics, which are less suitable for assessing the latent variables of interest (e.g. learning, performance, stochasticity) than more principled model-based analyses [44, 45]. In particular, computational models of learning are able to capture how performance evolves as a result of feedback, rather than simply assessing average performance [46]. Furthermore, previous work has suggested that behaviour in this learning task is modulated by three separate computational factors, which all contribute to the obtainable summary statistics: learning rates, unexplained biases in favour of one shape, and choice stochasticity [47].

We therefore fit reinforcement-learning models linked to stochastic choice models based on previous work using variants of this task [25–27]. Models were fit using Markov-Chain Monte-Carlo sampling. The models used and further methodological details of the model-fitting procedure can be found in the Supplementary Information.

Subsequently, we selected the model that (a) best fit the participants’ behaviour (according to the total integrated BIC score [48]), (b) showed good parameter recovery, and (c) was able to faithfully reproduce participant behaviour. Finally, we conducted statistical analyses on the relevant parameters from the best-fitting model.

#### Statistical analysis

The preregistered primary analysis of this study aimed to see if there were group differences in ability to alter learning rate between blocks, which we examined using a repeated-measures ANOVA on the difference between learning rates in blocks (sets of 80 trials) in which outcomes were volatile (see above: in block 1 both win and loss outcomes were volatile, and in blocks 2 and 3 win outcomes were volatile and loss outcomes stable, or vice versa, in an order counterbalanced between participants). We used counterbalance order and group as between-participant factors, and valence (win and loss learning rate adjustment) as the within-participant factor. We also analysed the learning rates in block 1, in which both outcomes were volatile, using an ANOVA with group and order as between-participant factors, and valence as a within-participant factor.

For completeness, we also explored the effects of group on the other parameters from the winning computational model, with between-subject factors of group and order, and within-subject factor of block.

### Pupillometry analysis

#### Preprocessing

Detailed information on the preprocessing of eye tracking data is provided in the supplementary materials. Notably, trials were excluded from analysis if >50% of the data in that trial was interpolated, and two participants who had >50% interpolation on >50% of the task trials were removed. One participant was removed as a power outage corrupted their pupillometry data.

After preprocessing, we had a time-series for each trial displaying pupil dilation to rewards, and to punishments, spanning 1 s before to 6 s after the outcome presentation. We subsequently calculated four mean time-series: for each combination of volatility and outcome (win volatile, win stable, loss volatile, and loss stable). As elsewhere, ‘block 1’ data was included in the volatile time-series. We then created a subtraction time-series from these: the difference between pupil dilation to receipt (subtracted from non-receipt) of volatile wins/losses minus stable rewards/losses.

#### Statistical analysis

We ran a cluster-based permutation mixed effects model using the ‘permutes’ package in R [49], with 1000 permutations and a random slope specified as (valence | id), to identify any time-points in which there were significant effects of group, valence, or interactions. The model had the following equation:

$${value} \sim {valence}* {Group}+\left({valence}|{id}\right),$$

where time was added as a continuous time-series variable. The package used shuffles the labels of the fixed effects, using a simplified version of the algorithm from Lee & Braun [50]. Note that the data included in the permutation test represent averaged time series for each individual for each valence. Subsequently, we ran post-hoc permutation linear mixed-effects models on relevant time-windows to obtain robust p-values.

## Results

### Participant characteristics

Demographic information and participant questionnaire scores (and group differences if found) are shown in Table 1.

### Switch/stay analyses do not discriminate between ED groups and healthy controls

**Fig. 2:**
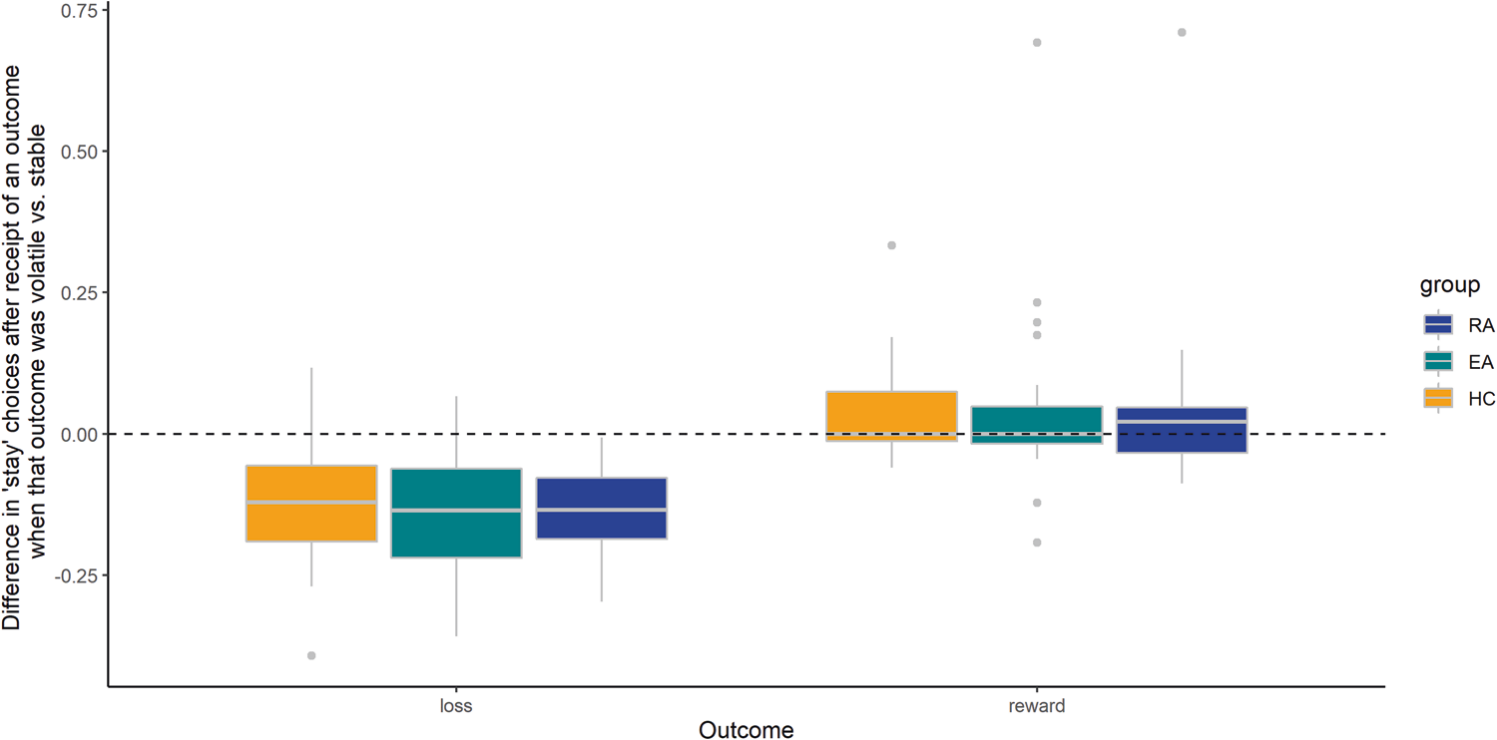
Difference in the mean proportion of times participants repeated a choice (‘stay’ choices) depending on whether the outcome they received was volatile (i.e. informative) or stable. These are shown using boxplots separated by group (colour) and by outcome (x-axis). The expected effect of block volatility would be numbers >0 for rewards and <0 for losses. Specifically, we expected to observe a greater tendency to ‘stay’ after the presentation of a reward in blocks where the ‘win’ outcomes are volatile, suggesting higher weighting of a recent ‘win’ than the previous long-run average of ‘win’ outcomes; alongside a greater tendency to switch in blocks where loss outcomes are volatile after the receipt of loss outcomes. This pattern can be seen in the figure. We expected to see this pattern reduced in the RA and EA groups, but in fact this pattern was not modified by group.

In a repeated-measures ANOVA, there were main effects of volatility (*F*_1,76_ = 16.21, *p* < 0.001) and valence (*F*_1,76_ = 364.60, *p* < 0.001), and an interaction effect between the two (*F*_1,76_ = 40.64, *p* < 0.001). This is an expected task effect: participants tended to ‘stay’ more when receiving a reward outcome when that reward was presented in a volatile block (*M* = 0.91, *SD* = 0.11) than when presented in a stable block (*M* = 0.87, *SD* = 0.18). Participants also tended to stay less when receiving a loss outcome when that loss was presented in a volatile block (*M* = 0.62, *SD* = 0.14) compared to a stable block (*M* = 0.75, *SD* = 0.16). There was not a main effect of group (*F*_2,76_ = 0.16, *p* = 0.851; Fig. 2) nor any interaction effects including group (group and volatility: *F*_2,76_ = 0.62, *p* = 0.542; group and valence: *F*_2,76_ = 0.04, *p* = 0.965; group, volatility and valence: *F*_2,76_ = 0.03, *p* = 0.971). There was an interaction effect between volatility, valence and order (*F*_1,76_ = 4.32, *p* = 0.041). The full results of this analysis can be seen in the Supplementary material.

At first sight, therefore, the different groups seem to show comparable learning rates (i.e., their tendency to switch or stay is not affected by group membership). In order to validate these results and explore the other computational factors that may govern behaviour in this task (overall choice stochasticity and unexplained preference biases), we modelled choice behaviour by linking different reinforcement learning and stochastic choice models.

### Best-fitting reinforcement learning model

We examined a set of reinforcement learning models which incorporated separate learning rates for rewards and losses, as in Pulcu & Browning [27] - see supplementary materials. The best-fitting reinforcement learning model (according to the integrated BIC [48]) had a single inverse temperature term, which captures choice stochasticity, or the extent to which participants do not act in accordance with the learnt values of the stimuli. We present results in the Supplementary Material which show that this model has good parameter recovery and was able to faithfully reproduce salient features of our participant data. We therefore use this model for subsequent inference.

### RA group show greater learning rate adjustment

**Fig. 3:**
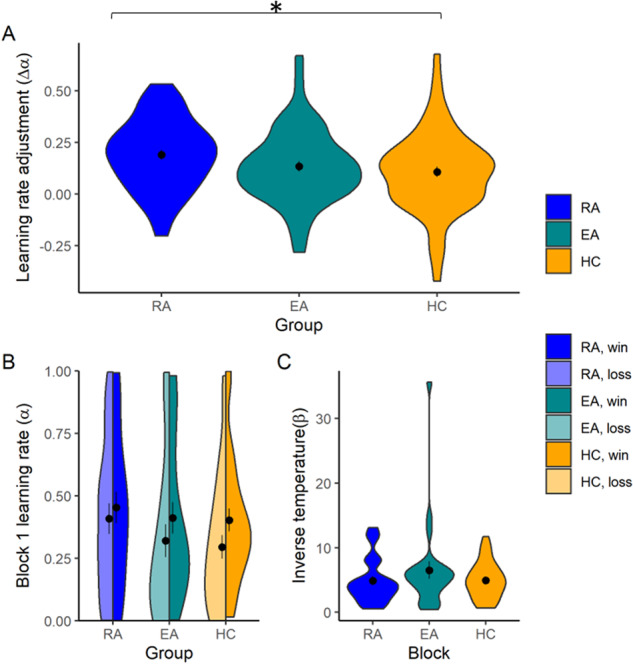
Learning rates, shown using violin plots, with mean and standard error shown using black dots and bars. **A** There was a significant effect of group on learning rate adjustment, such that the RA group adjusted their learning rate more than the HC group in response to volatility. **B** There was no group effect on the learning rate in block 1. **C** There was no group effect on inverse temperature, estimated across the task. *marks effects where *p* < 0.05.

We performed an ANOVA on the parameters from our best-fitting reinforcement-learning model, and found a significant group effect on the difference in learning rates between volatile and stable blocks (including block 1; *F*_2,76_ = 3.42, *p* = 0.038; Fig. 3A). This was driven by the RA group (groupwise RA vs. HC: *F*_1,53_ = 7.13, *p* = 0.010; groupwise EA vs. HC: *F*_1,53_ = 0.53, *p* = 0.468; groupwise RA vs. EA: *F*_1,46_ = 2.72, *p* = 0.106), who show elevated learning rate adjustment (*t*_110.78_ = 2.43, *p* = 0.017). When we examined the learning rates themselves in both the volatile block and the stable block, there was no effect of group (volatile block: *F*_2,76_ = 0.56, *p* = 0.574; stable block: *F*_2,76_ = 0.12, *p* = 0.888). Notably, there was no interaction between group and valence, so we do not investigate this further (*F*_2,76_ = 0.02, *p* = 0.985), nor any interaction between group and order (*F*_2,76_ = 0.91, *p* = 0.407). We also replicated a previous finding, of elevated win learning rate for wins when these are volatile compared to stable (*t*_161.65_ = 3.31, *p* = 0.001, *M* = 0.371 vs. 0.246), and the same for loss outcomes (*t*_161.57_ = 2.99, *p* = 0.003, *M* = 0.291 vs. 0.183). This demonstrates that, as expected, participants are adapting to volatility. The learning rate adjustment in all three groups was significantly different to 0 (RA: *t*_49_ = 8.00, *p* < 0.001, *M*(*sd*)=0.191(0.169); EA: *t*_49_ = 5.29, *p* < 0.001, *M(sd)*=0.134(0.180); HC: *t*_63_ = 4.43, *p* < 0.001, *M(sd)*=0.107(0.195)).

We also examined participants’ learning rates in block 1, in which both rewards and punishments were volatile. This block is particularly well-suited to the detection of baseline negative and positive biases in learning, as both of the outcomes are volatile and thus equally informative. In this exploratory analysis, we did not find a group effect (*F*_2,.79_ = 0.78, *p* = 0.463; Fig. 3B). There was also no effect of group on the other computational model parameter: inverse temperature (exploratory analysis: *F*_2,76_ = 1.04, *p* = 0.360; Fig. 3C).

### RA group also shows reduced effect of reward volatility on pupil dilation

**Fig. 4:**
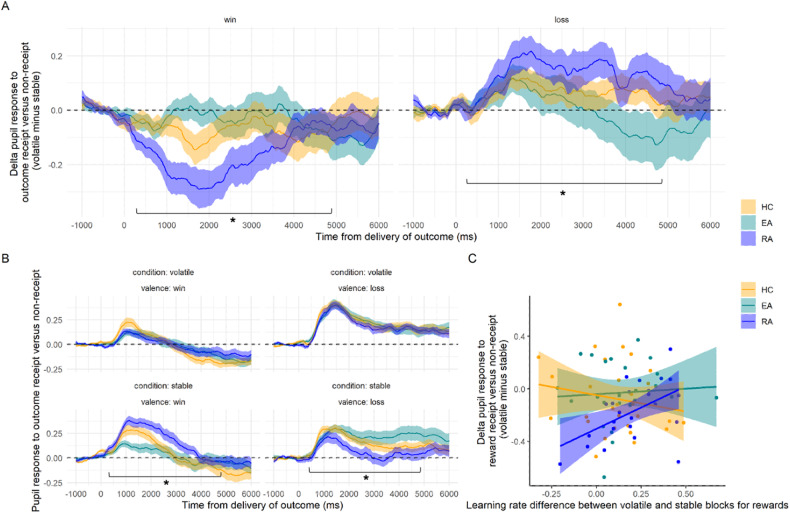
Results of pupillometry analysis. **A** Pupil dilation in response to the receipt of an outcome in blocks where that outcome is volatile, compared to blocks in which that outcome is stable. This plot is subdivided into pupil responses to rewards and losses. There was a significant interaction between group, condition (volatile vs. stable) and valence (win vs loss) from 342 ms to 4904 ms after outcome onset. The RA group showed reduced greater pupil dilation to wins in the stable condition (compared to both other groups), and greater pupil dilation to losses in the stable condition (compared to both other groups). The lines represent the mean, with the ribbon representing the standard error. **B** Results of pupillometry analysis, by condition and valence. Top left is the pupil response to loss outcomes when these are stable, top right win outcomes when these are stable, the bottom left is loss outcomes during blocks where losses are volatile, and the bottom right is win outcomes when they are volatile. The lines represent the mean, with the ribbon representing the standard error. **C** The overall volatile-stable pupil dilation response to rewards during the time of the significant group*valence effect was significantly correlated with behavioural learning rate difference in the RA group only. *marks significant cluster-mass statistic.

In our cluster-based permutation mixed-effects model, we observed significant clusters for all contrasts. In particular, we observed a significant interaction effect between group and valence, from 342 ms to 4904 ms after outcome presentation (*F*_2_ = 3.85, *p* < 0.001, see Supplementary Figure 6). We subsequently performed post-hoc exploratory analyses using permutation mixed-effects models on subsets of the data (separated back into four time courses) to identify the source of this effect. In the reward domain, there were main effects of group, condition, and an interaction effect; this was also true in the punishment domain. Further investigation showed no significant effect of group on pupil responses to volatile rewards (*F*_2_ = 0.01, *p* = 0.949), but there was a group effect in response to stable rewards (*F*_2_ = 2.73, *p* = 0.036), driven by the difference between RA and both other groups (vs. HC *F*_1_ = 3.26, *p* = 0.036, vs. EA *F*_1_ = 5.17, *p* = 0.029; Fig. 4C). There was no group effect when just including EA and HC (*F*_1_ = 0.38, *p* = 0.345). Similarly, there was no effect when examining volatile losses (*F*_2_ = 0.156, *p* = 0.682), but there was when including data for stable losses (*F*_2_ = 2.94, *p* = 0.022). This was driven by a significant difference between RA and EA (*F*_1_ = 4.86, *p* = 0.040); and RA and HC (*F*_1_ = 3.03, *p* = 0.043; Fig. 4B). There was no significant difference between EA and HC (*F*_1_ = 0.85, *p* = 0.185). The full results of these permutation tests can be observed in the Supplementary material.

### Learning rate adjustment and pupil volatility adjustment correlate in the RA group

The average of the pupil response to rewards, across the time period of the significant group*valence interaction, was positively correlated with learning rate adjustment for rewards in the RA group in an exploratory correlation analysis (*r*_22_ = 0.512, *p* = 0.011; Fig. 4C), but this was not true in any other group (EA: *r*_23_ = 0.067, *p* = 0.751; HC: *r*_28_ = −0.176, *p* = 0.351). We compared these correlation coefficients using a Fisher’s *r*-to-*z* transform, and found a significant difference between the RA and HC coefficients (*z* = 2.56, *p* = 0.011), but not between the RA and EA coefficients (*z* = 1.71, *p* = 0.087). There was no significant correlation between pupil response to losses and learning rate adjustment for losses in any group (*p*s > 0.2).

## Discussion

Contrary to our hypotheses, we found that a group of participants who had recovered from Anorexia Nervosa (RA group) showed greater learning rate adjustment when outcomes changed from volatile to stable (Fig. 3A). There was no difference between healthy control participants (HC) and those with high levels of symptoms but no diagnosed eating disorder (EA). In parallel, we found that the RA group showed greater pupil dilation to volatile vs. stable loss outcomes, soon after outcome delivery (Fig. 4B), and lower pupil dilation in response to volatile rewards compared to stable rewards soon after outcome delivery (Fig. 4B). These effects were driven by elevated pupil dilation to stable win outcomes, and reduced pupil dilation to stable loss outcomes. The pupil response difference for volatile win vs. stable win outcomes was positively correlated with learning rate adjustment in this group (Fig. 4C).

The finding that learning rate adjustment is greater in the RA group (Fig. 3A), is somewhat surprising, as much previous eating disorder research has focused on the trait of cognitive inflexibility as a possible marker for eating disordered behaviour [17, 18, 20–22]. Indeed, we hypothesised that we would observe the opposite result: that learning rate adjustment would be reduced. Reduced adjustment has been observed previously in anxiety disorders [27], and in those with high levels of internalizing symptoms [51], although findings in autism are similar to those we observe [52].

This result was specific to those in the RA group, and no differences were found between the EA group and HC group. This may reflect greater premorbid vulnerability to EDs: all of those in the RA group had a previously diagnosed eating disorder, whilst none of the EA group had a current ED diagnosis and were recruited purely on the basis of elevated scores on a symptom questionnaire (though some may be formally diagnosed in the future, but we do not know how many). They may thus not be an appropriate ‘risk’ group, and indeed, perhaps should be considered *resilient* to eating disorders, by virtue of the combination of elevated symptoms but no formal diagnosis. Importantly, the RA group included only those who had previously been diagnosed with one eating disorder – Anorexia Nervosa – whereas the EA group could include those with symptoms consistent with other eating disorders, so our finding may reflect different cognition between these disorders. Alternatively, it is possible that the EA group is an appropriate choice of risk group, but that differences in cognition are more subtle than would be observed in a group of participants with diagnosed eating disorders. The results presented in Fig. 3 suggest that perhaps this is the best explanation – this group have numerically greater learning rate adjustment values than the HC group, though less than the RA group. Future research should consider this, and use a more conservative estimate of effect sizes for power calculations for risk groups compared to clinically-diagnosed groups.

We also found an effect of group on the pupil dilation during volatile and relative to stable outcome receipt. This effect was driven by differences in the RA groups’ response to stable outcomes. Interestingly, the RA group shows greater pupil dilation to stable rewards and reduced pupil dilation to stable losses (compared to both other groups). This is further evidence that the RA group may be processing volatile outcomes unusually: in fact, the mean pupil dilation to stable rewards was greater than the mean pupil dilation to volatile outcomes (Fig. 4A). It may be significant that the effects are opposite for wins and losses – perhaps this reflects asymmetric processing of outcomes that are noisy but not volatile [53]. This type of imbalance was not, however, reflected either in the ‘baseline’ learning rates in response to the first block or by any effect of valence in the learning rate adjustment analyses. This discrepancy is not necessarily a contradiction: pupil dilation is thought to reflect many underlying computations, including volatility, but also surprise, salience, and mental effort [54, 55]. This discrepancy could thus be due to a fundamental between-groups difference in processing different types of uncertainty, or due to a mismatch in how participants from the different groups experience task difficulty or outcome salience. Future experiments should attempt to control for these other variables to further disentangle this effect. This pupil dilation time course for rewards was positively associated with learning rate adjustment, such that participants who adjusted their learning rates more (i.e., were further away from the HC group) showed more typical pupil dilation patterns (Fig. 4C), which is also consistent with the theory that greater pupil dilation reflects greater noradrenergic activity in response to increased volatility. This allowed us to link our model-based results (learning rate adjustment) with a model-free, physiological measure (pupil dilation). Speculatively, this may be a marker of improved cognitive flexibility: individuals who recover from eating disorders adjust their learning rates further, and their pupil responses are closer to those observed in the control group. It is also surprising that there is no correlation between learning rate adjustment and pupil dilation within the other groups studied, or in the loss domain, as has been observed previously [25].

Previous cognitive neuroscience studies have shown that changes in pupil diameter may reflect a number of different influences, from cognitive effort, to uncertainty, to surprise, to changes in the world [29, 31]. Further work could attempt to manipulate these independently in different ED groups to ascertain which is reflected in our findings. Importantly, various noradrenergic agents such as propanalol [34] or atomoxetine [35] may be able to alter responses to volatility. Above, we note that noradrenaline has been implicated in EDs using measurements of noradrenaline metabolites [38–40], with emerging evidence that atomoxetine may be effective in eating disorders featuring binging behaviours [36, 37]. Notably, however, our RA group (in whom we observed pupillometry differences) were not selected for high levels of binging, which may suggest that noradrenaline differences may be more broadly present across ED groups. In light of our findings, experimental medicine studies or early-stage clinical trials of noradrenergic compounds in other, non-binging eating disorders may be a promising new avenue for exploration.

### Limitations

This study has several limitations. Firstly, ‘expected’ or ‘irreducible’ uncertainty was not manipulated independently from volatility, and may have its own effects on behaviour and pupil dilation [23, 24, 53]. Specifically, expected uncertainty is high when an outcome is probabilistic rather than deterministic, and is maximal when the probabilities of all outcomes are equiprobable – as was the case in our ‘stable’ blocks, where outcomes were associated with stimuli with 50% probability. This may mean that our results relate to differences in uncertainty estimation more generally, rather than volatility. Future work should use a task in which other versions of uncertainty are held stable whilst volatility is manipulated. On a related note, both outcomes were always volatile in the first block. This design choice allowed us to check for the existence of baseline differences in learning rate. Given that no differences were observed, in future research it might be more beneficial to have a fully randomised block order to avoid any ‘priming’ with expectations of high volatility, and to balance the number of times that participants would be expected to increase (compared to decrease) their learning rates.

Secondly, we did not recruit any individuals who were currently unwell with eating disorders. Part of our rationale for this was to ensure that malnutrition and underweight status did not drive results in this cognitively-demanding task. However, we therefore cannot claim our findings are necessarily representative of those with a current eating disorder.

## Conclusions

In conclusion, we find evidence for differences in the processing of outcome volatility between a group who had recovered from Anorexia Nervosa, and healthy control participants. The RA group showed greater adjustment of their learning rates to volatility than controls. In this same group, we also observed an atypical lower pupil dilation to volatile than stable outcomes, particularly in those participants who showed learning rate adjustment closest to control participants. Importantly, given these findings, manipulation of noradrenaline levels using pharmacological agents may be an interesting future direction for eating disorder research.

## Disclaimer

The views expressed are those of the authors and not necessarily those of the NHS, the NIHR or the Department of Health.

### Supplementary information

Supplementary material

## Data Availability

Open data and code are not available as not all participants consented to have their data shared, even if they could not be identified after anonymisation.
