# Supplementary material for: Adaptive learning from outcome contingencies in eating-disorder risk groups
Source: Transl Psychiatry. 2023 Nov 4;13:340. doi: 10.1038/s41398-023-02633-w (PMC10625579; doi:10.1038/s41398-023-02633-w)
Supplement: Supplementary file 1 — Supplementary material [file 41398_2023_2633_MOESM1_ESM.docx]

Supplementary materials

**Pike, Alexandra C.*^1,2,3,4^, Sharpley, Ann L.^3,4^, Park, Rebecca J.^3,4^, Cowen, Philip J.^3,4^, Browning, Michael^+ 3,4^, Pulcu, Erdem^+ 3,4^**

# Supplementary Methods

## Participants

There were no a priori estimates of the likely effect size in this study, as a previous study which related performance on this task to a clinical measure used a correlational analysis rather than a case-control design (1). However, we performed a sensitivity analysis, and based on our chosen sample size of 25 per Eating Disorder (ED) group and 32 control participants, we determined that we would have 0.95 power to detect an effect size of *d* = 0.89.

### Inclusion and exclusion criteria

All participants were aged between 18 and 45, fluent in English, and had a Body-Mass Index (BMI) of over 18.5kg/m^2^ (measured during the study visit). All participants were also screened for current psychiatric diagnoses using the Structured Clinical Interview for the Diagnostic and Statistical Manual of Mental Disorders, Fifth Edition (DSM-5), Research Version (SCID-5-RV). Those who appeared to have a current psychiatric diagnosis that would be likely to interfere with the results of the study were excluded (e.g. current depressive episode, current generalised anxiety disorder, current obsessive-compulsive disorder, or a current substance use disorder). Participants were also excluded if they were taking a psychoactive medication such as an anti-depressant. Participants did not smoke more than 5 cigarettes per day, and the number of cigarettes they did smoke (if applicable) was recorded.

### Group definitions

The Recovered from Anorexia Nervosa (RA) group was defined by a self-reported former diagnosis of Anorexia Nervosa (AN; reported as being made by a healthcare professional), confirmed by a SCID-5-RV interview indicating the presence of former AN, and no current ED diagnosis. They (as was true for all participants) were required to have a BMI of over 18.5kg/m^2^, and to self-report as being recovered for over a year. Their current psychological recovery status was indexed in terms of the Eating Disorder Examination Self-Report Questionnaire (2), with full psychological recovery defined by a score of less than 1 standard deviation above the global mean, based on the norms. This was recorded but not used as an exclusion criterion.

The group who were concerned about their eating, shape and weight were defined by a score of above 20 on the Eating Attitudes Test (EAT-26; 3). This is the putative clinical cut-off proposed by the original authors, and indicates significant concerns about shape, weight and eating, and corresponding behaviours.

The control group was required to score less than 1 standard deviation above the global mean on the EDE-Q, below 16 on the Clinical Impairment Assessment for EDs (CIA; which measures impairment from ED-related symptoms) (4), and below 20 on the EAT-26.

## Ethical Approval

This study was approved by the University of Oxford’s Central University Research Ethics Committee, with reference R51898. All participants read an information sheet prior to participation and gave written informed consent.

## Task Presentation

The volatility task was presented on a VGA monitor connected to a laptop computer running Psychopy version 1.85.2. Participants were encouraged to keep their head still, and to assist in this they rested on a head-and-chin rest placed 42cm from the screen. An eye-tracking system was mounted on the rest, (Eyelink 1000 Plus; SR Research, Ottawa, Canada). The eye-tracking device was set up to record from both eyes (binocularly): it recorded participants’ gaze coordinates and pupil area, at a rate of 500Hz. A fixation cross was presented in the centre of the screen. Either side were the abstract shapes, offset by about 9 degrees visual angle. Below the fixation cross the ‘total money’ was displayed, and updated at the start of each new trial to reflect the outcomes from the last. The ‘win’ and ‘loss’ outcomes were presented separately in a random order, and were displayed for a jittered interval of 2-6 (mean 4) seconds. Sounds were played when the ‘win’ outcome appeared by the shape chosen by the participant (‘cha-ching’ sound), and when the ‘loss’ outcome appeared by the shape (‘buzz’ sound).

## General statistical approach

Wherever applicable we used a Greenhouse-Geisser correction to adjust for lack of sphericity. To further clarify significant effects from the mixed ANOVAs, we used post-hoc Welch’s t-tests, which conservatively assume unequal variance between groups.

## Reinforcement learning models

The models that we fit to the data included parameters that have been used before in other work using this task or variants of it (1,5,6), but in addition we also incorporated models that use sensitivity parameters, to examine whether our data could be best explained by individual differences in the valuation of the outcomes. We made two specific assumptions when choosing which model space to explore. We chose to only fit models with two learning rate parameters, based on previous evidence that participants performing this task learn separately from win and loss outcomes (6); and we chose to fit separate learning rates and bias parameters to each block of the task, with all other parameters estimated across all blocks, based on evidence showing that participants are able to flexibly alter their learning rate as volatility changes, but no other parameters vary (6). These assumptions were tested by examining the fit of a model that was the ‘mirror-image’ of the best-fitting model in our model space – i.e. a model with a single learning rate, and other parameters that were divided by valence and block (6).

Notably, for all of these models we used fictive updating (i.e. both the chosen and unchosen option are updated), as the rewards and punishments that would have been obtained had the participant chosen the other stimulus are displayed on screen, such that participants had full information about the potential outcomes of that trial.

### Learning models

The learning models we fit to this data are all variants of Q-learning reinforcement models (7). In all the models we considered, based on previous evidence (5,6) we included two separate learning rates (α), for reward and loss outcomes, which govern the extent to which the value of a given stimulus A (Q_A_) is updated by the prediction error (δ), such that separate Q values are learnt and maintained for win (*Equation 1*) and for loss (*Equation 2*) for each stimulus:

|  | $Q_{A,t+1}^{win}= Q_{A,t}^{win}+ \alpha_{win}*\delta_{A,t}^{win}$ | *Equation 1* |
| --- | --- | --- |

|  | $Q_{A,t+1}^{loss}= Q_{A,t}^{loss}+ \alpha_{loss}*\delta_{A,t}^{loss}$ | *Equation 2* |
| --- | --- | --- |

Where

|  | $\delta_{A,t}^{win}={win}_{A.t}-Q_{A,t}^{win}$ | *Equation 3* |
| --- | --- | --- |

|  | $\delta_{A,t}^{loss}={loss}_{A,t}-Q_{A,t}^{loss}$ | *Equation 4* |
| --- | --- | --- |

Some models also contained one or more sensitivity parameters (ρ), which act as a multiplier on the value of the reward or punishment received by the participant and thus alter the value of the prediction error (*Equation 5*):

|  | $\delta_{A,t}=\rho*{outcome}_{A,t}-Q_{A,t}$ | *Equation 5* |
| --- | --- | --- |

It is possible to have more than one sensitivity parameter (*Equation 6* and *Equation 7* show the prediction errors for wins and losses when separate win and loss sensitivities are used):

|  | $\delta_{A,t}^{win}=\rho_{win}*{win}_{A,t}-Q_{A,t}^{win}$ | *Equation 6* |
| --- | --- | --- |
|  | $\delta_{A,t}^{loss}=\rho_{loss}*{loss}_{A,t}-Q_{A,t}^{loss}$ | *Equation 7* |

### Action models

These learning models play the role of updating the values associated with different stimuli on a trial-by-trial basis. Link functions are then used to convert these values into choices or actions, as, in general, participants do not simply choose the option with the higher value, but may explore, or show stochastic behaviour. The simplest link function (*Equation 8*) includes a single inverse temperature parameter (β), and is a logit function over the Q-values for both stimuli (denoted A and B):

|  | $P_{A,t}=\frac{\exp\left( Q_{A,t}*\beta\right)}{\exp\left( Q_{A,t}*\beta\right)+\exp\left( Q_{B,t}*\beta\right)}$ | *Equation 8* |
| --- | --- | --- |
| where | ${Q_{A,t}=Q}_{A,t}^{win}-Q_{A,t}^{loss}$ |  |

We also tested models with two inverse temperature parameters, one for win and one for loss, which were multiplied by the win and loss Q values before they were entered into the softmax (*Equation 9*):

|  | $Q_{A,t}=Q_{A,t}^{win}*\beta_{win}-Q_{A,t}^{loss}*\beta_{loss}$ | *Equation 9* |
| --- | --- | --- |

Notably, the inverse temperature parameter in softmax link functions captures both sensitivity and choice stochasticity, and thus including inverse temperature and sensitivity in the same model results in poor recovery. Therefore, for models with sensitivity terms, we include a link function with a lapse term (ξ) instead (*Equation 10*):

|  | $P_{A,t}=\frac{\exp\left( Q_{A,t} \right)}{\exp\left( Q_{A,t} \right)+ \exp\left( Q_{B,t} \right)}*\left( 1-\xi\right)+\frac{\xi}{2}$ | *Equation 10* |
| --- | --- | --- |

We have also previously observed unexplained biases in favour of one stimulus in participants’ choice behaviour (8) and so we also tested models which incorporated this bias as an additive term to the Q values of one stimulus (*Equation 11*), and a subtractive term to the Q values of the other (*Equation 12*), just before these are added into the softmax.

|  | $Q_{A,t}=Q_{A,t}+bias$ | *Equation 11* |
| --- | --- | --- |

|  | $Q_{B,t}=Q_{B,t}-bias$ | *Equation 12* |
| --- | --- | --- |

Combining these learning and action models resulted in 8 models, shown in Supplementary Table 1 below. To preview our results, the best-fitting model was model number 1, marked with an asterisk.

| Model number | Model | Learning rate | Sensitivity | Inverse Temperature | Lapse | Bias |
| --- | --- | --- | --- | --- | --- | --- |
| 1* | 2lr1b | α_win_ and α_loss_ |  | β |  |  |
| 2 | 2lr1b1bias | α_win_ and α_loss_ |  | β |  | Bias |
| 3 | 2lr2b | α_win_ and α_loss_ |  | β_win_ and β_loss_ |  |  |
| 4 | 2lr2b1bias | α_win_ and α_loss_ |  | β_win_ and β_loss_ |  | Bias |
| 5 | 2lr1s1lapse | α_win_ and α_loss_ | ρ |  | ξ |  |
| 6 | 2lr1s1lapse1bias | α_win_ and α_loss_ | ρ |  | ξ | Bias |
| 7 | 2lr2s1lapse | α_win_ and α_loss_ | ρ_win_ and ρ_loss_ |  | ξ |  |
| 8 | 2lr2s1lapse1bias | α_win_ and α_loss_ | ρ_win_ and ρ_loss_ |  | ξ | Bias |

Supplementary Table 1: Models fit to choice data on the volatility task. The Greek letters correspond to parameters as highlighted in the sections above, but in brief: α_win_ and α_loss_ represent separate learning rates for wins and losses. ρ represents sensitivity, which can also be divided into separate parameters for win and loss. β represents inverse temperature, which can also be separated by win and loss. ξ is a lapse parameter. Note that models 1, 3, 5 and 7 were used in (6), in which model 7 was the winning model; and models incorporating a bias term are inspired by (9), in which the winning model was equivalent to model 6. The best-fitting model is marked using an asterisk.

### Blockwise-parameters

For all the models specified above, we assumed that learning rates could be different in each block, due to the between-block volatility manipulations, in line with previous work (5,6), but that inverse temperature, lapse and sensitivity did not change between blocks. In particular, learning rate is hypothesised to change as volatility does: elevated learning rates imply that more recent outcomes are weighted more heavily than outcomes that are further back in time, which allows a participant to respond appropriately to high volatility (new unexpected events update learnt values more highly). There is a strong reason to believe bias might also differ in each block - a new pair of stimuli are presented in each block, and thus a preference for one stimulus should not carry over to the next block. It should be noted that this is a bias for a particular stimulus, not a response key or side of the screen - in this case, it might be more reasonable to presume a bias persists across the entire task. There is no a priori reason to suppose that other parameters should change within each block, as the volatility manipulation and stimulus change should not affect how stochastic or noisy participants are (beta, lapse) or how sensitive they are to rewards and punishments (the values of which are held constant). We explicitly tested this assumption by comparing our winning model with one in which the learning rates did not vary between blocks, and other parameters did, and found that it fit the data less well (see Results section).

### Model fitting

We fit these models to the data using Markov-Chain Monte-Carlo sampling implemented in R v4.0.2 and RStan v2.19.3. We used 4 chains for all model fitting, with half of the iterations used for warmup, and half for sampling. For model comparison, we used 2000 iterations per chain, and for final inference, we used 10,000 iterations per chain. The sampling algorithm used was No-U-Turn Sampling (NUTS) (10). We used a Bayesian, non-hierarchical approach to estimating parameters, which, despite worse recovery than hierarchical estimation, does not risk either inflating or deflating the effect size resulting from either choosing to use separate priors for each group or a single overall prior (11). We estimated the hyperparameters (i.e. the sufficient statistics of each parameter) simultaneously with the participant-level parameters. We used a Phi-approx transformation (an approximation to the Phi distribution, the inverse cumulative density function of the standard normal distribution) to bound parameter values between 0 and 1 for both learning rates and lapse parameters. We used a square transform to ensure all sensitivity and inverse temperature parameters were greater than 0, and a tanh transform to ensure all bias parameters were between -1 and 1. The hyperparameters from which parameters were drawn were given by a standard Normal distribution (0,10).

## Pupillometry Analysis

### Preprocessing

Data obtained using the Eyelink 1000 Plus system was preprocessed as in Pulcu & Browning (2017). This involved the following steps. Firstly, blinks were identified using the system’s built-in filter and removed. Secondly, a low-pass Butterworth filter with a cut-off of 3.75Hz was applied. The data was then z-transformed across the whole task (6,12). Then, the pupil dilation response to win and loss outcomes was extracted separately based on the time they appeared in each trial (which outcome appeared first was randomised). The time-window that was extracted for each outcome was from 1s before until 6s after outcome presentation. This was then baseline corrected, by subtracting the mean of the 1s before the outcome was presented (the baseline) from each timepoint within the time window. We interpolated when blinks or saccades to outside the area caused us to remove datapoints. Individual trials were excluded from analysis if more than 50% of the data in that trial was interpolated (1,6), and participants were removed if more than 50% of their trials were removed for this reason (n=2). Another participant had to be removed as the end of their pupillometry data was lost due to a power outage. For the remainder of the participants, the mean percentage of trials excluded was 7%. As in the previous studies using this task, the first 10 trials from each block were not included in analysis, as initial pupil adaptation is likely in response to luminance changes during this period (1,12).

These steps result in two sets of time series for each participant: one set including the portion during each trial corresponding to the display of the ‘win’ outcome, and one set including the portion during each trial corresponding to the display of the ‘loss’ outcome. Subsequently, for both win and loss outcomes, we separated the timeseries into two: one based on the pupil response when the outcome was received (i.e. when that outcome was associated with the shape they had chosen), and one based on the pupil response when the outcome was not received (i.e. when that outcome was not associated with the shape they had chosen).

These timeseries were then further separated into blocks, such that for each participant, we had a mean time series for pupil dilation to win and loss, given either receipt or non-receipt of the outcome. The difference between the corresponding chosen and unchosen time-series was then calculated, leading to a timeseries for each outcome of pupil dilation in response to receipt vs. non-receipt of that outcome (i.e. a timeseries for chosen-unchosen loss outcomes). Finally, we created a timeseries for the difference in pupil dilation between volatile and stable conditions (across all blocks) to receipt (vs. non-receipt) of outcomes.

# Supplementary Results

## No group difference on measures of accuracy

There was no significant effect of either group or order on participants’ overall task performance (as measured by the money they won):

Response: money_won

       Effect    df  MSE    F   ges p.value

1       group 2, 76 5.97 0.14  .004    .868

2       order 1, 76 5.97 0.07 <.001    .794

3 group:order 2, 76 5.97 2.36  .059    .101

---

Signif. codes:  0 ‘***’ 0.001 ‘**’ 0.01 ‘*’ 0.05 ‘+’ 0.1 ‘ ’ 1

Similarly, there was no significant effect of group or order on participants’ overall task performance as measured by how many choices they made that aligned with those a Bayesian Ideal Observer (defined as in previous work: 6) would make:

Anova Table (Type 3 tests)

Response: bio

       Effect    df  MSE      F   ges p.value

1       group 2, 76 0.01   0.21  .006    .808

2       order 1, 76 0.01   0.05 <.001    .830

3 group:order 2, 76 0.01 2.38 +  .059    .099

---

Signif. codes:  0 ‘***’ 0.001 ‘**’ 0.01 ‘*’ 0.05 ‘+’ 0.1 ‘ ’ 1


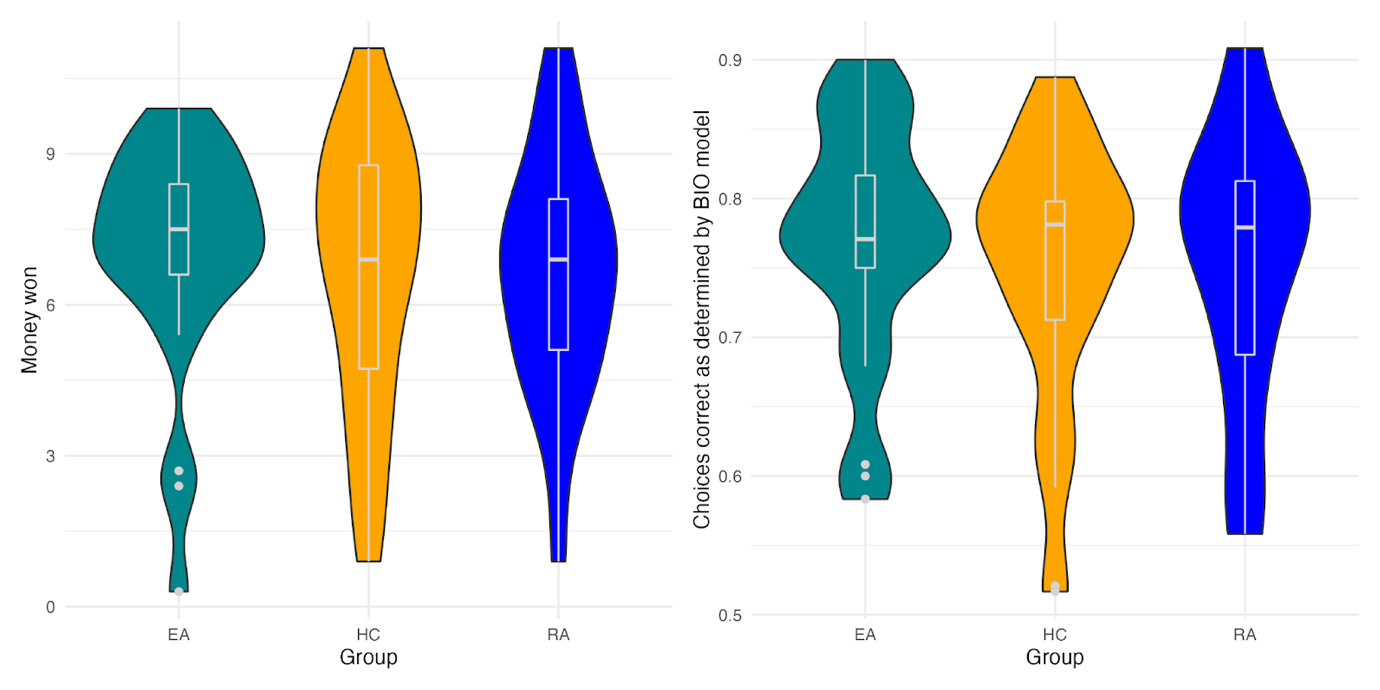


Supplementary Figure 1: Violin plots with embedded box plots showing participants’ performance on some key metrics: A) how much money each participant won (all participants started with £1.50 and each win gained them 15p, and each loss reduced their total by 15p), and B) how many of their choices were predicted by a Bayesian Ideal Observer model.

## Best-Fitting Reinforcement Learning Model

### Integrated BIC comparison

We calculated the integrated BIC for each model fit to participant choice data. The integrated BIC uses the posterior distribution of the likelihood (which can be estimated using Stan), and reflects ‘goodness-of-fit’ to the data, but penalises for model complexity using a term containing the number of parameters (here, we estimated the effective number of parameters using the ‘loo’ package in R, which accounts for parameter covariance or information pooling). Results of comparing integrated BIC values for our set of models with two learning rates are shown in Supplementary Figure 2A, and results of comparing the best-fitting of these models with an equivalent model in which learning rate is constant, and inverse temperature varies by block and valence, are displayed in Supplementary Figure 2B.


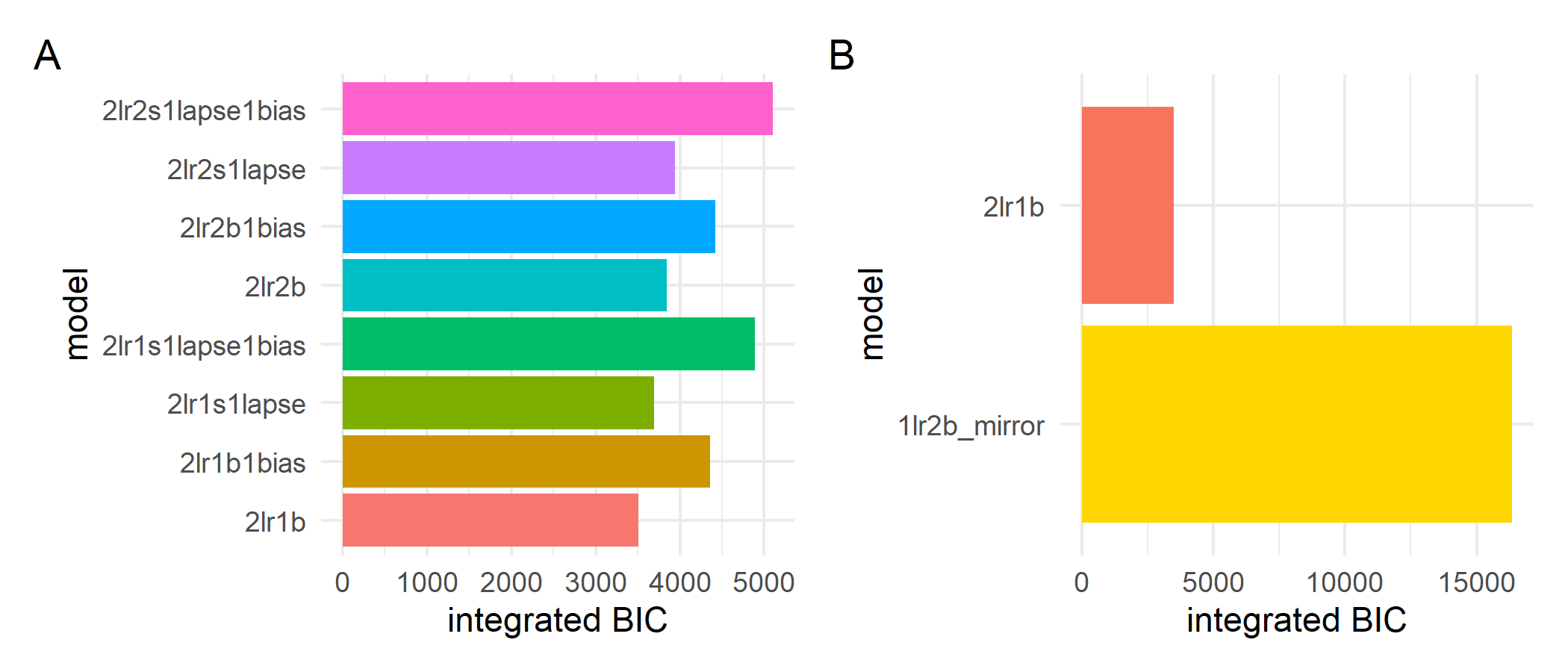


Supplementary Figure 2: Model comparison using integrated BIC scores. Note that lower integrated BIC score indicates a better fit to the data. A) Set of models with two learning rates, B) a comparison between the best-fitting model and an equivalent one in which learning rate is constant across blocks, but inverse temperature varies by outcome valence and block.

### Parameter recovery

We assessed how well parameters generated from realistic distributions could be recovered by fitting our best-fitting model: one with two learning rates and one inverse temperature (Supplementary Figure 2). Learning rates were generated from a *Beta*(1,1) distribution, and inverse temperature parameters were generated from a *Gamma*(3,0.5) distribution. Parameter estimation was performed using a non-hierarchical model run in RStan v.2.26.3 using four chains, each with 1000 warmup iterations and 1000 sampling iterations. All parameters were recovered with a high degree of accuracy (matrix diagonal), and there was no notable tradeoff between parameters (the off-diagonal).


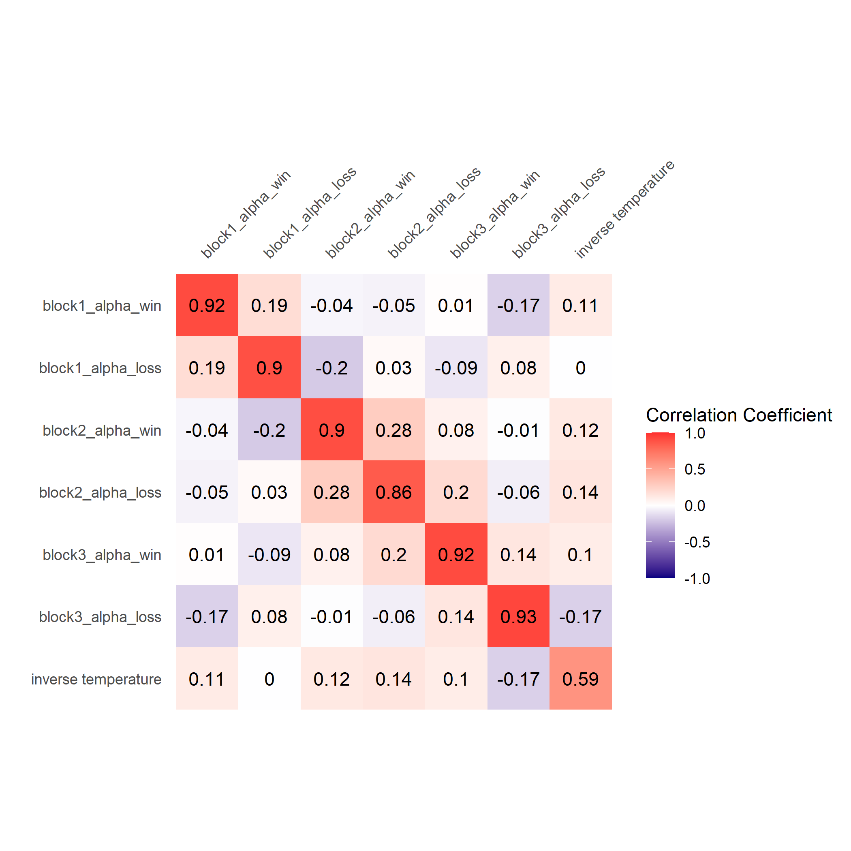


Supplementary Figure 3: Parameter recovery for the best-fitting model. The diagonal shows the correlation coefficient between parameters generated from realistic distributions and those estimated by the model; and the off-diagonal shows the correlations between these estimated parameters and other estimated parameters. A high value on the diagonal indicates high fidelity recovery, and a low value on the off-diagonal indicates minimal tradeoff between different parameters as estimated by our model-fitting procedure.

### Reproduction of behaviour

Here, we assessed how the model-generated choices captured the key model-free metrics, including switch-stay behaviour shown in Figure 2 of the main text.

#### Money won

We correlated the ‘money won’ by participants and the estimate of ‘money won’ generated by the winning model. The amount of money won as a result of participants’ actual choices and as a result of model-simulated choices were highly correlated: *r*_80_=0.901, *p*<0.001 (Supplementary Figure 4A).

#### Proportion predicted

We also analysed the proportion of choices that were correctly predicted by the winning model. In an ANOVA with group and order as between-subject factors, there was no main effect of group (Supplementary Figure 4B; *F*_2,76_ = 0.25, *p*=0.777). There was also no main effect of order (*F*_1,76_ = 0.22, *p*=0.637) or interaction effect between group and order (*F*_2,76_ = 1.76, *p*=.180).

These analyses suggest that the model does a good job of capturing the key features of the data – analyses on the model-predicted choices revealed the same patterns of choice behaviour, and accuracy was high in predicting an overall performance variable (money won), along with the specific sequence of choices.

#### Switch-stay analysis by block and group

We analysed the proportion of ‘stay’ choices (i.e. the participant chooses the same stimulus as on the preceding trial) after receiving an outcome (reward or loss), when that outcome was either volatile or stable. We also included group, order, and choice source (original choices vs. model-predicted choices). This is an extension of the analysis reported in the main paper, with an initial factor of choice source. Proportion was logit transformed. The general pattern found in Figure 2 was replicated (Supplementary Figure 4C), but there was a significant main effect of data source (*F*­­­_1,76_ = 246.91, *p*<.001), such that stay probability was estimated to be lower by the model than the proportions found in the actual choice data. As in the actual choice data, there was a main effect of valence (*F*_1,76_ = 446.81, *p*<.001) and an interaction between volatility and valence (*F*_1,76_ = 75.76, *p* < .001), and a significant interaction between valence and source, and volatility and source, and valence, volatility and source. There was, however, no main effect of group (*F*_2,76_ = 0.28, *p* = .757) or any interaction effect including group, so we conclude that this model should not introduce any group-specific bias.


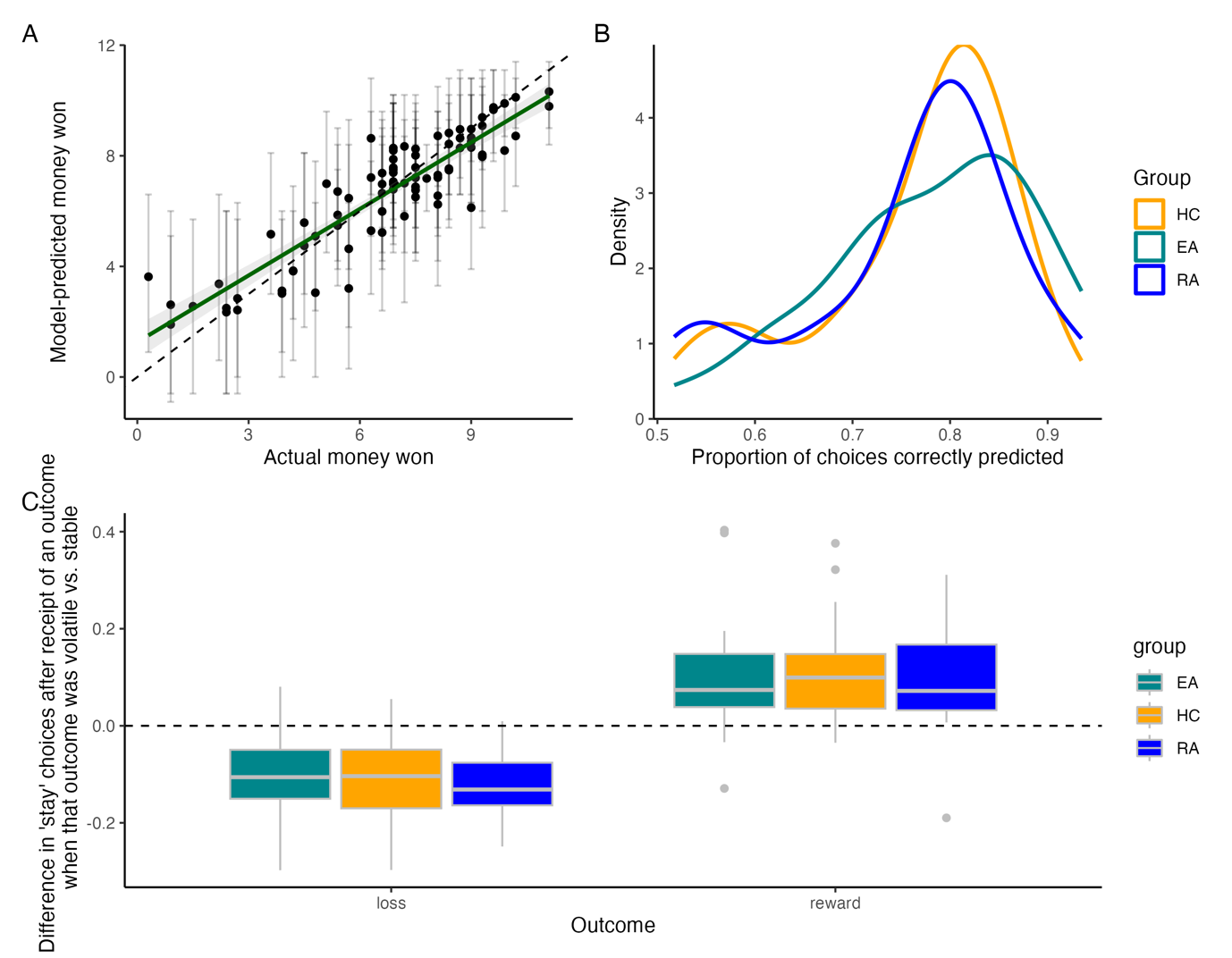


Supplementary Figure 4: Comparison of model-predicted and actual behavioural data. A) Relationship between actual money won and model-predicted money won. Error bars represent 95% Bayesian Credible Intervals, extracted from the posterior predictions of participant choices. The green line represents the line of best fit, using a linear regression of predicted money won against actual money won. The dashed line represents the line y=x, corresponding to instances where the predicted and actual money won by participants were identical. B) Smoothed kernel density estimate of proportion of choices correctly predicted by the model, out of 20,000 generated choices per trial per participant. This is a continuous version of a histogram. C) The difference in the mean proportion of times participants repeated a choice (‘stay’ choices) depending on whether the outcome they received was volatile (i.e. informative) or stable, for the model-predicted choices. These are shown using boxplots separated by group (colour) and by outcome (x-axis). The reader can compare this to Figure 2 in the main paper.

## Tabulated results for behavioural analyses

Switch/stay analysis

Anova Table (Type 3 tests)

Response: logit_stay

Effect df MSE F ges p.value

1 order 1, 76 2.92 0.28 .002 .597

2 group 2, 76 2.92 0.16 .003 .851

3 order:group 2, 76 2.92 1.72 .030 .186

4 valence 1, 76 0.63 364.60 *** .412 <.001

5 order:valence 1, 76 0.63 0.02 <.001 .877

6 group:valence 2, 76 0.63 0.04 <.001 .965

7 order:group:valence 2, 76 0.63 0.52 .002 .597

8 volatility 1, 76 0.34 16.21 *** .016 <.001

9 order:volatility 1, 76 0.34 0.69 <.001 .409

10 group:volatility 2, 76 0.34 0.62 .001 .542

11 order:group:volatility 2, 76 0.34 0.10 <.001 .903

12 valence:volatility 1, 76 0.45 40.64 *** .052 <.001

13 order:valence:volatility 1, 76 0.45 4.32 * .006 .041

14 group:valence:volatility 2, 76 0.45 0.03 <.001 .971

15 order:group:valence:volatility 2, 76 0.45 0.46 .001 .633

---

Signif. codes: 0 ‘***’ 0.001 ‘**’ 0.01 ‘*’ 0.05 ‘+’ 0.1 ‘ ’ 1

**Learning rate analysis**

All groups

Anova Table (Type 3 tests)

Response: value

               Effect    df  MSE      F   ges p.value

1               group 2, 76 0.03 3.42 *  .034    .038

2               order 1, 76 0.03   1.66  .008    .202

3         group:order 2, 76 0.03   0.91  .009    .407

4             valence 1, 76 0.04   0.28  .002    .599

5       group:valence 2, 76 0.04   0.02 <.001    .985

6       order:valence 1, 76 0.04   0.01 <.001    .907

7 group:order:valence 2, 76 0.04   0.26  .004    .771

---

Signif. codes:  0 ‘***’ 0.001 ‘**’ 0.01 ‘*’ 0.05 ‘+’ 0.1 ‘ ’ 1

RA vs HC

Anova Table (Type 3 tests)

Response: value

               Effect    df  MSE      F   ges p.value

1               group 1, 53 0.02 7.13 *  .045    .010

2               order 1, 53 0.02   0.08 <.001    .773

3         group:order 1, 53 0.02   0.11 <.001    .741

4             valence 1, 53 0.05   0.24  .003    .625

5       group:valence 1, 53 0.05   0.01 <.001    .918

6       order:valence 1, 53 0.05   0.24  .003    .628

7 group:order:valence 1, 53 0.05   0.05 <.001    .825

---

Signif. codes:  0 ‘***’ 0.001 ‘**’ 0.01 ‘*’ 0.05 ‘+’ 0.1 ‘ ’ 1

EA vs HC

Anova Table (Type 3 tests)

Response: value

               Effect    df  MSE      F   ges p.value

1               group 1, 53 0.03   0.53  .003    .468

2               order 1, 53 0.03 2.81 +  .018    .100

3         group:order 1, 53 0.03   1.12  .007    .295

4             valence 1, 53 0.05   0.18  .002    .672

5       group:valence 1, 53 0.05   0.03 <.001    .873

6       order:valence 1, 53 0.05   0.05 <.001    .833

7 group:order:valence 1, 53 0.05   0.22  .003    .638

---

Signif. codes:  0 ‘***’ 0.001 ‘**’ 0.01 ‘*’ 0.05 ‘+’ 0.1 ‘ ’ 1

RA vs EA

Anova Table (Type 3 tests)

Response: value

Effect df MSE F ges p.value

1 group 1, 46 0.03 2.72 .029 .106

2 order 1, 46 0.03 1.34 .014 .254

3 group:order 1, 46 0.03 1.42 .015 .240

4 valence 1, 46 0.03 0.15 .002 .703

5 group:valence 1, 46 0.03 0.00 <.001 .945

6 order:valence 1, 46 0.03 0.00 <.001 .994

7 group:order:valence 1, 46 0.03 0.68 .007 .415

---

Signif. codes: 0 ‘***’ 0.001 ‘**’ 0.01 ‘*’ 0.05 ‘+’ 0.1 ‘ ’ 1

#### Tabulated results for volatile vs. stable

There was no significant effect including the ‘group’ term in either the stable or volatile condition. The only significant effects were of valence (volatile: *F*(1,76) = 8.06, *p*= 0.006; stable: *F*(1,76)=4.27, *p*=0.042. Further analysis by valence indicated no remaining significant effects, as can be seen in the tables below.

Win volatile

Response: value

       Effect    df  MSE    F  ges p.value

1       group 2, 76 0.06 0.45 .012    .638

2       order 1, 76 0.06 1.01 .013    .319

3 group:order 2, 76 0.06 0.29 .008    .747

Loss volatile

Response: value

       Effect    df  MSE    F   ges p.value

1       group 2, 76 0.06 0.55  .014    .580

2       order 1, 76 0.06 0.00 <.001    .961

3 group:order 2, 76 0.06 0.91  .023    .406

Win stable

Response: value

       Effect    df  MSE    F  ges p.value

1       group 2, 76 0.07 0.13 .003    .880

2       order 1, 76 0.07 0.16 .002    .687

3 group:order 2, 76 0.07 0.42 .011    .656

Loss stable

Anova Table (Type 3 tests)

Response: value

       Effect    df  MSE    F  ges p.value

1       group 2, 76 0.05 0.17 .004    .848

2       order 1, 76 0.05 0.61 .008    .439

3 group:order 2, 76 0.05 1.08 .028    .345

## Is there an effect of anxiety?

In a mixed model with a random intercept, there was no significant effect including trait anxiety - either in the whole sample, or in the HC group alone. Detailed results are shown in the Table below:

Whole group

Type III Analysis of Variance Table with Satterthwaite's method

                            Sum Sq   Mean Sq NumDF DenDF F value Pr(>F)

order                    0.0026934 0.0026934     1   156  0.0781 0.7803

STAI.trait               0.0011807 0.0011807     1   156  0.0342 0.8535

valence                  0.0014251 0.0014251     1   156  0.0413 0.8392

order:STAI.trait         0.0169307 0.0169307     1   156  0.4909 0.4846

order:valence            0.0000259 0.0000259     1   156  0.0008 0.9782

STAI.trait:valence       0.0003409 0.0003409     1   156  0.0099 0.9209

order:STAI.trait:valence 0.0002074 0.0002074     1   156  0.0060 0.9383

HC only

Type III Analysis of Variance Table with Satterthwaite's method

                           Sum Sq  Mean Sq NumDF DenDF F value Pr(>F)

order                    0.037655 0.037655     1    56  0.9488 0.3342

STAI.trait               0.025846 0.025846     1    56  0.6512 0.4231

valence                  0.012199 0.012199     1    56  0.3074 0.5815

order:STAI.trait         0.033623 0.033623     1    56  0.8472 0.3613

order:valence            0.046053 0.046053     1    56  1.1604 0.2860

STAI.trait:valence       0.021363 0.021363     1    56  0.5383 0.4662

order:STAI.trait:valence 0.057927 0.057927     1    56  1.4596 0.2321

When trait anxiety is included as a covariate in the main analysis, there is a significant 3 way interaction between trait anxiety, group, and valence: (*F*=3.48, *p=*0.0336). Further post-hoc analysis shows that this is limited to ‘win’ outcomes, and seems to be driven by a different direction of relationship in the RA group compared to the others (see graph below and results tables).

Analysis including anxiety and eating-disorder group

Type III Analysis of Variance Table with Satterthwaite's method

Sum Sq Mean Sq NumDF DenDF F value Pr(>F)

group 0.039143 0.019571 2 140 0.5914 0.55490

order 0.000011 0.000011 1 140 0.0003 0.98521

STAI.trait 0.000635 0.000635 1 140 0.0192 0.89005

valence 0.006158 0.006158 1 140 0.1861 0.66684

group:order 0.054970 0.027485 2 140 0.8306 0.43793

group:STAI.trait 0.053247 0.026623 2 140 0.8046 0.44934

order:STAI.trait 0.000070 0.000070 1 140 0.0021 0.96341

group:valence 0.169447 0.084723 2 140 2.5603 0.08089 .

order:valence 0.013006 0.013006 1 140 0.3930 0.53172

STAI.trait:valence 0.014334 0.014334 1 140 0.4332 0.51152

group:order:STAI.trait 0.068066 0.034033 2 140 1.0285 0.36024

group:order:valence 0.118787 0.059394 2 140 1.7949 0.16995

group:STAI.trait:valence 0.230193 0.115096 2 140 3.4782 0.03356 *

order:STAI.trait:valence 0.026034 0.026034 1 140 0.7867 0.37661

group:order:STAI.trait:valence 0.166133 0.083067 2 140 2.5103 0.08490 .

Win only

Analysis of Variance Table

Response: value

Df Sum Sq Mean Sq F value Pr(>F)

group 2 0.08664 0.043318 1.2828 0.28368

order 1 0.01609 0.016091 0.4765 0.49228

STAI.trait 1 0.00161 0.001614 0.0478 0.82759

group:order 2 0.06855 0.034274 1.0150 0.36767

group:STAI.trait 2 0.27616 0.138078 4.0892 0.02091 *

order:STAI.trait 1 0.00499 0.004991 0.1478 0.70182

group:order:STAI.trait 2 0.19786 0.098928 2.9298 0.05999 .

Residuals 70 2.36367 0.033767

---

Signif. codes: 0 ‘***’ 0.001 ‘**’ 0.01 ‘*’ 0.05 ‘.’ 0.1 ‘ ’ 1

Correlations

| Group | Correlation coefficient | p-value |
| --- | --- | --- |
| RA | 0.5346195 | 0.0059 |
| EA | -0.1634835 | 0.4349 |
| HC | -0.1724045 | 0.3454 |

Note that a Fisher’s r-to-z transform analysis indicates that the difference in correlation between RA and HC is significant (two-tailed p-value: 0.00641), as is the difference in correlation between RA and EA (p=0.0115). The difference in correlation between the EA and HC groups was not significant (0.974).


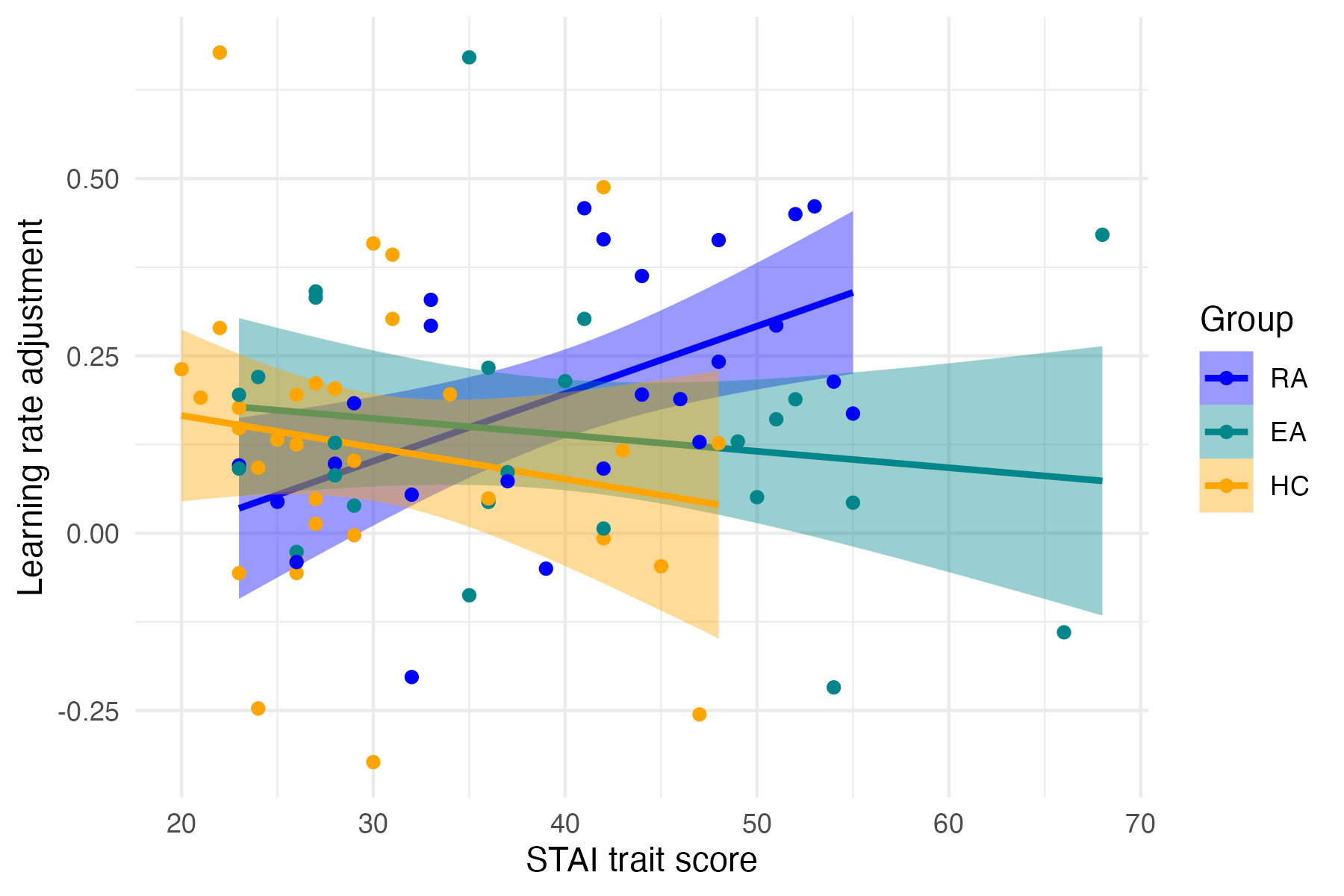


Supplementary Figure 5: The interaction between trait anxiety (STAI-trait scale) and learning rate adjustment for win outcomes only. Note that this relationship differs between groups.

## Pupillometry analysis

### Results of permutation tests

The results of a cluster-based permutation test specified as

clusterperm.lmer(value ~ valence * Group + (valence | id), data=delta_df,series.var=~time,parallel=TRUE, nperm=1000, type='anova') are shown in **Supplementary Figure 6.**


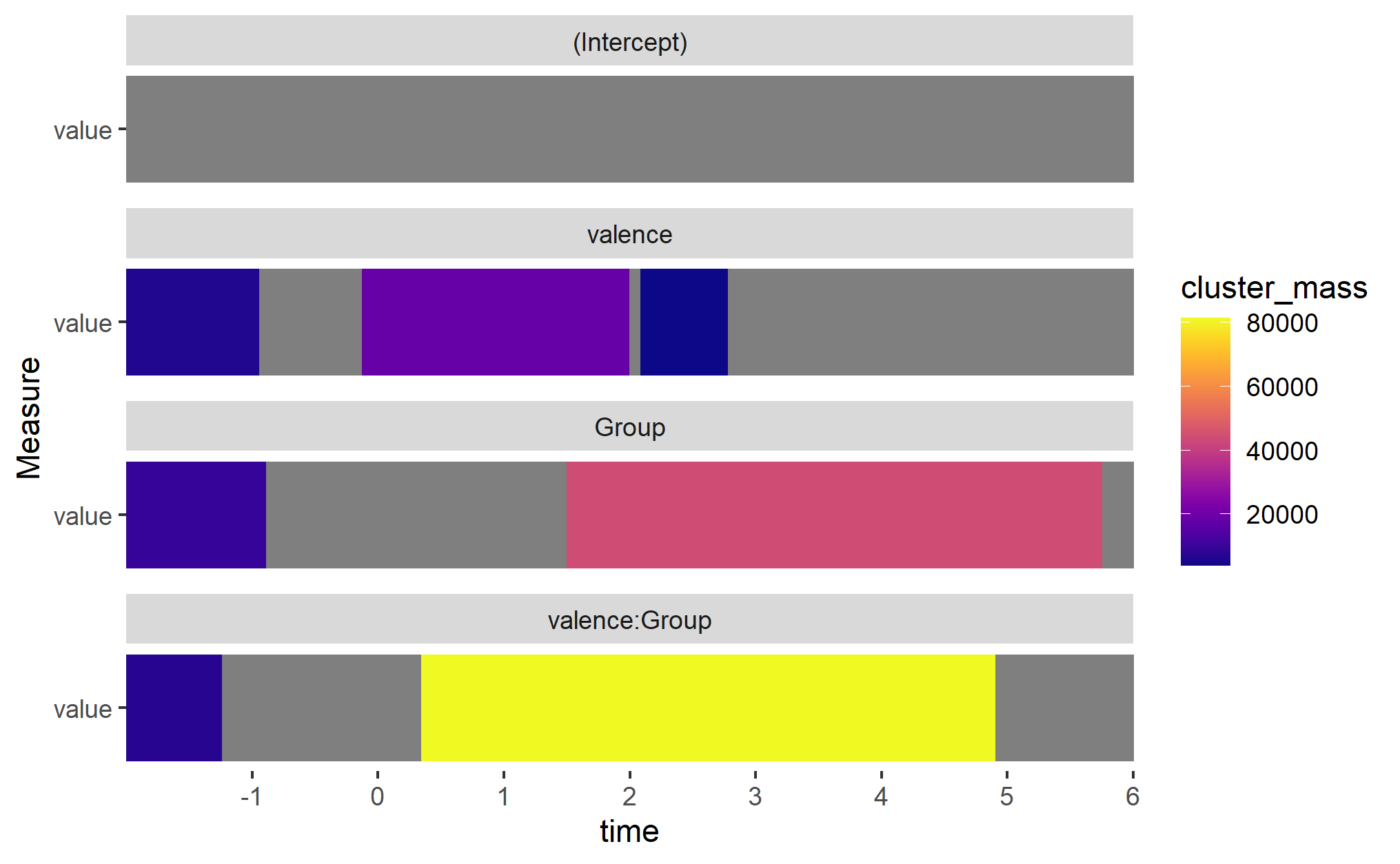


Supplementary Figure 6: Displays significant cluster mass statistics over time from a permutation test of pupil dilation. Notably, there is a significant valence:Group interaction. Time=0 represents the onset of the outcome presentation, and the previous second is the baseline used to baseline-correct time series. Data was recorded up to 6s post stimulus presentation.

Overall permutation test

|  |
| --- |

| **Factor** | **df** | **LRT** | **F** | **p** |
| --- | --- | --- | --- | --- |
| (Intercept) | 1 | 0.1189104 | 0.01810727 | NA |
| valence | 1 | 1.7193887 | 0.26315320 | 0.182 |
| Group | 2 | 17.1021545 | 1.76300320 | 0.010 |
| valence:Group | 2 | 32.8645514 | 3.84710419 | 0.000 |

Rewards

|  |
| --- |

| **Factor** | **df** | **LRT** | **F** | **p** |
| --- | --- | --- | --- | --- |
| (Intercept) | 1 | 23.829784 | 4.698214 | NA |
| Group | 2 | 15.155608 | 3.447701 | 0.000 |
| condition | 1 | 11.956697 | 2.268810 | 0.009 |
| Group:condition | 2 | 7.654125 | 1.763168 | 0.013 |

Rewards in the volatile condition

| **Factor** | **df** | **LRT** | **F** | **p** |
| --- | --- | --- | --- | --- |
| (Intercept) | 1 | 0.005186385 | 0.001894784 | NA |
| Group | 2 | 0.069477213 | 0.014317752 | 0.949 |

Rewards in the stable condition

|  |
| --- |

| **Factor** | **df** | **LRT** | **F** | **p** |
| --- | --- | --- | --- | --- |
| (Intercept) | 1 | 9.577023 | 3.719645 | NA |
| Group | 2 | 6.058273 | 2.729596 | 0.036 |

Rewards in the stable condition: RA vs HC

| **Factor** | **df** | **LRT** | **F** | **p** |
| --- | --- | --- | --- | --- |
| (Intercept) | 1 | 7.211853 | 4.127692 | NA |
| Group | 1 | 5.772672 | 3.259373 | 0.036 |

Rewards in the stable condition: EA vs HC

|  |
| --- |

| **Factor** | **df** | **LRT** | **F** | **p** |
| --- | --- | --- | --- | --- |
| (Intercept) | 1 | 6.0296646 | 3.3495640 | NA |
| Group | 1 | 0.7154561 | 0.3785145 | 0.345 |

Rewards in the stable condition: RA vs EA

|  |
| --- |

| **Factor** | **df** | **LRT** | **F** | **p** |
| --- | --- | --- | --- | --- |
| (Intercept) | 1 | 1.590823 | 0.7912917 | NA |
| Group | 1 | 9.569300 | 5.1715759 | 0.029 |

Loss condition

| **Factor** | **df** | **LRT** | **F** | **p** |
| --- | --- | --- | --- | --- |
| (Intercept) | 1 | 89.658006 | 22.043093 | NA |
| Group | 2 | 15.917056 | 3.879539 | 0.000 |
| condition | 1 | 9.763622 | 1.839710 | 0.016 |
| Group:condition | 2 | 6.016356 | 1.470596 | 0.034 |

Loss in the volatile condition

|  |
| --- |

| **Factor** | **df** | **LRT** | **F** | **p** |
| --- | --- | --- | --- | --- |
| (Intercept) | 1 | 92.1972933 | 63.8548842 | NA |
| Group | 2 | 0.5808728 | 0.1556772 | 0.682 |

Loss in the stable condition

|  |
| --- |

| **Factor** | **df** | **LRT** | **F** | **p** |
| --- | --- | --- | --- | --- |
| (Intercept) | 1 | 36.17519 | 16.761544 | NA |
| Group | 2 | 6.12341 | 2.949997 | 0.022 |

Loss in the stable condition: RA vs HC

|  |
| --- |

| **Factor** | **df** | **LRT** | **F** | **p** |
| --- | --- | --- | --- | --- |
| (Intercept) | 1 | 28.459078 | 20.045335 | NA |
| Group | 1 | 5.389678 | 3.032169 | 0.043 |

Loss in the stable condition: EA vs HC

|  |
| --- |

| **Factor** | **df** | **LRT** | **F** | **p** |
| --- | --- | --- | --- | --- |
| (Intercept) | 1 | 25.211047 | 16.8110441 | NA |
| Group | 1 | 1.601556 | 0.8541856 | 0.185 |

Loss in the stable condition: RA vs EA

|  |
| --- |

| **Factor** | **df** | **LRT** | **F** | **p** |
| --- | --- | --- | --- | --- |
| (Intercept) | 1 | 30.815388 | 20.994590 | NA |
| Group | 1 | 9.050107 | 4.864327 | 0.04 |

# Supplementary References

1. Browning M, Behrens TE, Jocham G, O’Reilly JX, Bishop SJ (2015): Anxious individuals have difficulty learning the causal statistics of aversive environments. *Nat Neurosci* 18: 590–596.

2. Fairburn CG, Beglin SJ (1994): Assessment of eating disorder psychopathology: Interview or self-report questionnaire. *International Journal of Eating Disorders* 16: 363–370.

3. Garner DM, Olmsted MP, Bohr Y, Garfinkel PE (1979): About the Eating Attitudes Test - The Eating Attitudes Test (EAT-26). *Http://Www.Eat-26.Com/Screening.Php*. pp 1–23.

4. Bohn K, Fairburn CG (2008): The Clinical Impairment Assessment Questionnaire (CIA 3.0). *Cognitive Behaviour Therapy and Eating Disorders*. New York: Guilford Press.

5. Behrens TEJ, Woolrich MW, Walton ME, Rushworth MFS (2007): Learning the value of information in an uncertain world. *Nat Neurosci* 10: 1214–1221.

6. Pulcu E, Browning M (2017): Affective bias as a rational response to the statistics of rewards and punishments. *eLife* e27879.

7. Sutton, R.S; Barto, A. G. (1998): *Introduction to Reinforcement Learning*, vol. 135. Cambridge: MIT Press.

8. Pulcu E, Browning M (2019): The Misestimation of Uncertainty in Affective Disorders. *Trends in Cognitive Sciences* 23: 865–875.

9. Pulcu E, Shkreli L, Holst CG, Woud ML, Craske MG, Browning M, Reinecke A (2019): The Effects of the Angiotensin II Receptor Antagonist Losartan on Appetitive Versus Aversive Learning: A Randomized Controlled Trial. *Biological Psychiatry* 86: 397–404.

10. Hoffman MD, Gelman A (2011): The No-U-Turn Sampler: Adaptively Setting Path Lengths in Hamiltonian Monte Carlo. *arXiv* 1111: 5.

11. Valton V, Wise T, Robinson OJ (2020): *The Importance of Group Specification in Computational Modelling of Behaviour*. PsyArXiv. https://doi.org/10.31234/osf.io/p7n3h

12. Nassar MR, Rumsey KM, Wilson RC, Parikh K, Heasly B, Gold JI (2012): Rational regulation of learning dynamics by pupil-linked arousal systems. *Nat Neurosci* 15: 1040–1046.
